# Influence of Light on Nutrition

**Published:** 1881-01

**Authors:** 


### INFLUENCE OF LIGHT ON NUTRITION.

The influence of light upon the nutrition of the animal body is a subject which appears to have received more attention from the practical physician than from the pysiologist—at least in this country. There are few persons who do not believe in the hygienic value of abundant light as well as of fresh air; but it is difficult to point to other than pathological or negative evidence in favor of this opinion. We have now at our command definite physiological facts respecting the influence of light upon the excretion of carbonic acid in the lower animals. These are the results of investigations carried out by Moleschott and Fubini, (*Centralblatt, f. d., Med. Wiss.*, 1880, page 562). By carefully arranged experiments these observers were able to estimate, not only the comparative, but the absolute or quantitative effect of light upon amphibia, birds, and mammals, and to exclude from the result the effects of temperature and movement. The result was in all classes of animals so far the same—the admission of light to an animal which had been previously kept in the dark raising the amount of carbonic acid excreted from 20 to 40 per cent., and from 11 to 27 per cent. even when the animal had been previously blinded. It thus appears, as we should have expected, that light affects nutrition not only through the retina, but also through the skin. What is more remarkable is that the nutrition of the muscular system, after removal of the skin, central nervous system, and eyes, was found to be 50 per cent. more active in light than in darkness; while the brain and spinal cord similiarly tested showed similar, though less marked, nutritive activity under the influence of light. Further experiments showed that blue-violet light exercises precisely the same influence on nutrition as mixed (white) light; while red light affects frogs similarly to darkness, although it stimulates nutrition to a moderate degree in birds and mammals. The individual tissues proved to be affected by colored light in much the same way as the entire body.

